# Plasmacytoid dendritic cells in dermatology^☆^^☆☆^

**DOI:** 10.1016/j.abd.2020.08.006

**Published:** 2020-11-21

**Authors:** Natasha Favoretto Dias de Oliveira, Claudia Giuli Santi, Celina Wakisaka Maruta, Valeria Aoki

**Affiliations:** Department of Dermatology, Faculty of Medicine, Universidade de São Paulo, São Paulo, SP, Brazil

**Keywords:** Adaptive immunity, Dendritic cells, Dermatological diseases, Dermatology, Innate immunity

## Abstract

Plasmacytoid dendritic cells are part of the dendritic cells family and are a relevant link between innate and adaptive immunity. They are the most potent producers of type 1 interferon, generating antiviral response, stimulating macrophages and dendritic cells and inducing activation and migration of natural killer cells. Plasmacytoid dendritic cells also exert a role as antigen-presenting cells, promote T-lymphocyte responses, immunoregulation, plasma cells differentiation and antibody secretion. Even though plasmacytoid dendritic cells are not usually present in normal skin, their presence is detected in healing processes, viral infections, and inflammatory, autoimmune, and neoplastic diseases. In recent years, the presence of plasmacytoid dendritic cells in several dermatological diseases has been described, enhancing their potential role in the pathogenesis of such conditions. Future studies on the role of plasmacytoid dendritic cells in dermatology may lead to new therapeutic targets.

## Introduction

Plasmacytoid dendritic cells (pDCs) are part of dendritic cells family and develop from hematopoietic stem cells in the bone marrow. Lennert and Remmele first mentioned this then new cell type in 1958, detected in the paracortical region of reactive lymph nodes and named them lymphoblasts^1^; since the late 1980s, pDCs received several denominations over time (plasmacytoid T-cells, plasmacytoid monocytes).^2^, ^3^, ^4^, ^5^

Characteristically, pDCs exhibit alpha chain receptor interleukin-3 (CD123) and blood derived dendritic cell antigen-2 (BDCA-2 or CD303), and are key mediators of innate immunity; they are the most potent producers of type 1 interferon (IFN) (IFN-a, IFN-b, IFN-l, IFN-w, and IFN-t), secreting a thousand times more IFN-α and IFN-β than other cell types.^6^

pDCs are activated through Toll-like receptors (TLRs) 7 and 9, starting a signal that induces the expression of multiple immunomodulatory and pro-inflammatory molecules encoding genes, such as IFN-α. TLR7 responds to single-stranded RNA (rich in guanosine or uridine) found in viruses (as influenza and respiratory syncytial virus), while TLR 9 detects single-stranded DNA molecules containing unmethylated CpG motifs (CpG ODN) commonly found in virus genome like herpes simplex (HSV). Thus, pDCs detect viral infections through the recognition of viral nucleic acids and are important mediators of antiviral immunity.^6^

**Figure 1:**
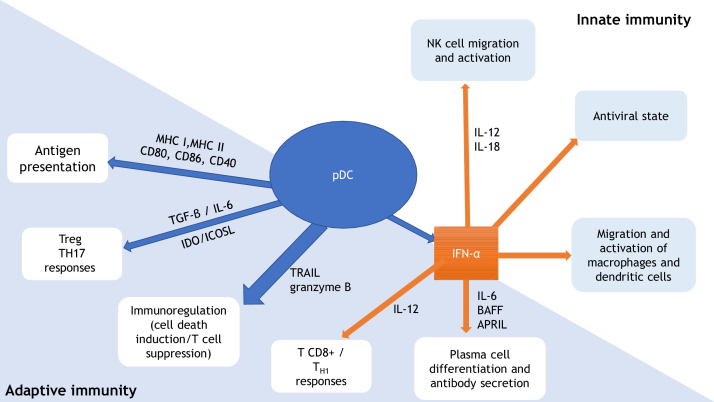
Plasmacytoid dendritic cells (pDCs): relevant link between innate and adaptive immunity. Adapted from Mitchell et al., 2018 and Swiecki et al., 2015.^9^, ^10^ pDC, plasmacytoid dendritic cell; MHC, major histocompatibility complex; CD, cluster of differentiation; Treg, T-regulatory lymphocyte; TH, T-helper lymphocyte; TGF-β, transforming growth factor beta; IL, interleukin; IDO, indoleamine-2,3-dioxygenase; ICOSL, inducible costimulator ligand; TRAIL, tumor necrosis factor-related apoptosis-inducing ligand; IFN-α, interferon alpha; BAFF, B cell-activating factor of the TNF family; APRIL, a proliferation-inducing ligand; NK, natural killer.

pDCs are a relevant link between innate and adaptive immunity (Fig. 1). They produce type 1 IFN in response to viral infections, promoting an antiviral state through IFN-stimulated genes expression and infected cells apoptosis. In addition to IFN, interleukin (IL)-12 and IL-18 activate natural killer (NK) cells, leading to IFN-γ secretion and lysis of target cells. IFN production by pDCs also induce macrophage and dendritic cells stimulation. Through expression of major histocompatibility complexes (MHC) and co-stimulatory molecules (as CD80, CD86, CD40), pDCs promote adaptive immunity by acting as antigen-presenting cells for both CD4+ and CD8+ T-lymphocytes. pDC expression of IDO (indoleamine-2,3-dioxygenase) and ICOSL (inducible costimulatory ligand) lead to T-regulatory (Treg) response, while pDCs production of TGF-β and IL-6 promotes T-helper (Th) 17 commitment. Production of IFN-I and IL-12 by the pDCs stimulates TCD8+ activity and polarization of TCD4+ cells into Th1. pDCs production of IFN-I, IL-6, B cell-activating factor of the TNF family (BAFF), and a proliferation-inducing ligand (APRIL) lead to expansion and stimulation of antibody-producing plasma cells. TNF-related apoptosis-inducing ligand (TRAIL) and granzyme B give pDCs the capacity to kill tumor cells, induce apoptosis of infected TCD4+ cells, and suppress T-cell proliferation. In addition, pDCs themselves stimulate plasmacytoid cells differentiation, through type 1 IFN and IL-6.^6^, ^7^, ^8^, ^9^, ^10^

pDCs are not usually present in normal skin; they infiltrate the skin in healing processes and in several conditions, such as viral infections, inflammatory, autoimmune, and neoplastic diseases.^2^, ^3^ Review of the literature regarding the presence of pDCs and dermatologic diseases are below described.

## Cutaneous lupus erythematosus

In lupus erythematosus (LE), pDCs are the main producers of IFN-α. Circulating pDCs are known to be reduced in patients with systemic lupus erythematosus (SLE), since they migrate and infiltrate target tissues, such as the skin. Several studies have shown large amounts of IFN-α producing pDCs in chronic discoid lupus lesions.^11^, ^12^, ^13^

Tomasini et al. found differences in pDCs' distribution among different types of lupus: superficial expression in skin specimens of subacute lupus and discoid lupus, and distribution around deep vessels in cases of tumid lupus and multicentric reticulohistiocytosis.^3^ They also found a positive relation between intensity of the inflammatory infiltrate and amount of pDCs.

Additionally, the number of pDCs in LE and dermatomyositis (DM) lesions directly correlated with the expression of myxovirusresistance protein (MxA), a protein induced by IFN-α/β, suggesting that pDCs are an important IFN-α source in such diseases.^11^, ^14^, ^15^ McNiff et al. verified that pDCs were preferably detected at the epidermis in patients with dermatomyositis when compared to patients with lupus, where the location was mainly in the dermis, suggesting that in LE circulating immune complexes migrate through the vessels before skin deposition.^11^

## Lichen planus

Vries et al. observed abundant CD123+ pDCs in lichen planus (LP) skin fragments, arranged the band-like lymphocytic infiltrate at the dermo-epidermal junction. In addition, they demonstrated replication of herpes virus 7 in plasmacytoid dendritic cells in active LP lesions, followed by its decrease in clinical remission.^16^, ^17^

Wang et al. described increased plasmacytoid dendritic cells infiltration in oral LP, proposing them as mediators of the innate immune response in this disease.^18^

## Lichen striatus

Badr et al. found pDCs in the inflammatory infiltrate of lichen striatus (LS), suggesting that these cells play a role in the pathogenesis of the disease. Additionally, there is a perieccrine distribution of pDCs in LS, which may be a useful criterium to histologically differentiate LS from LP.^19^

## Atopic dermatitis

In patients with atopic dermatitis (AD), there was an increase in circulating pDCs in comparison to controls; moreover, recruitment of pDCs in AD lesions was much lower than the amount found in other dermatoses, such as LE and psoriasis. The poor recruitment of pDCs, resulting in reduced production of type 1 IFN in AD lesions, could explain enhanced susceptibility of patients with AD to viral infections, such as herpes simplex or poxvirus infections.^20^, ^21^

## Polymorphic light eruption

Rossi et al., in contrast to previous studies, demonstrated significant increase of dermal pDCs in patients with polymorphic light eruption when compared to healthy controls, probably due to the photo provocation testing.^22^, ^23^

## Psoriasis

Nestle et al. found large amounts of pDCs in the dermal T-lymphocyte infiltrate of psoriatic plaques, absent in the skin of healthy donors and AD patients.^24^ Additionally, pDCs were also found in the skin adjacent to the plaques, even without apparent lesions. They also demonstrated reduction in pDCs in the peripheral blood of patients with psoriasis when compared to healthy individuals, suggesting that the accumulation of pDCs in the skin of patients with psoriasis occurs due to the redistribution pDCs from the blood to the skin.^24^ In animal models, the authors demonstrated production of IFN-α by pDCs in the injured skin, and its influence on the

Another study in psoriasis showed that heliotherapy provided not only clinical improvement, but also reduction of pDCs number and expression of MxA, a marker for IFN-α in lesional skin, strengthening the role of pDCs in psoriasis.^25^

Chemerin is a chemotactic factor for serum pDCs. Albanesi et al. demonstrated a strong expression of chemerin and the presence of pDCs in the dermis of skin adjacent to psoriatic lesions.^26^ There was a low expression of chemerin and few pDCs in chronic psoriatic plaques, suggesting that the expression of chemerin occurs in the early stages of psoriasis lesions and is temporally correlated with the presence of pDCs.

## Pityriasis lichenoides

The pathophysiology of pityriasis lichenoides (PL) is not fully understood. MxA expression was demonstrated in PL lesions, establishing local production of type 1 IFN.^27^, ^28^

Karouni et al. demonstrated the presence of pDCs in the skin of PL patients in both forms, pityriasis lichenoides et varioliformis acuta (PLEVA) and pityriasis lichenoides chronica (PLC).^29^ Furthermore, they found intense and diffuse expression of MxA, demonstrating its activation and production of type 1 IFN. The authors suggest that viral infections, autoantigens, trauma, antigens against drugs and tumors – previously associated with cases of PL – could also contribute to the recruitment and activation of pDCs, as demonstrated in other interface dermatitis, such as LP, LS, LE, and DM.^29^

## Graft vs. host disease

Malard et al. showed the presence of pDCs in skin fragments of patients with acute graft-versus-host disease (GVHD), as well as a strong expression of MxA, signaling local production of type 1 IFN.^30^ Previously, the same group reported a reduction in serum levels of pDCs in patients with acute GVHD, suggesting pDCs' infiltration into the GVHD target tissues.^30^, ^31^ However, the presence of CD123+ cells in the oral mucosa of patients with chronic GVHD was not superior when compared to controls in the study carried out by Botari et al., which suggests an early participation of pDCs in GVHD.^32^

## Warts

The demonstration of the presence of activated pDCs in inflamed viral warts was based on the intense expression of MxA, contrary to the data by Tomasini et al.^3^, ^33^ The opposite results may be related to the different histological criteria for inflamed warts used by the two groups.^33^

Tassone et al. evaluated patients with WHIM syndrome (warts, hypogammaglobulinemia, infections, and myelokathexis), who have refractory warts; the authors pointed out reduction in pDCs in peripheral blood and in skin lesions, and absence of type 1 IFN production, assessed through the expression of MxA, suggesting inability of antiviral activity of pDCs *via* TLR9.^34^

## Fungal diseases

Pagliari et al. evaluated the role of pDCs in three deep mycoses (chromoblastomycosis [CBM], lobomycosis, and paracoccidioidomycosis [PCM]).^35^ pDCs were found in 37% of the skin fragments of patients with CBM and in 50% of specimens of patients with PCM, but were absent in patients with lobomycosis. The authors hypothesize that pDCs would be a secondary source of cytokines, relevant to antifungal activity in cases of CBM and PCM. In lobomycosis, the absence of pDCs implies that such are not involved in the antifungal immune response.

## Leprosy

Massone et al. described the absence of CD123 expression in skin biopsy fragments of leprosy patients, except for focal expression in two cases of erythema nodosum leprosum, implying in noninvolvementof pDCs in the immune response against *Mycobacterium leprae.*^36^ Hirai et al., on the other hand, found pDCs in the inflammatory infiltrate and around the vessels in the skin of leprosy patients, emphasizing enhanced number of pDCs in tuberculoid leprosy, when compared to lepromatous leprosy.^37^

## Skin tumors

The presence of pDCs in skin tumors has been evaluated by several authors. Abbas et al. identified the presence of pDCs in 100% of cases of keratoacanthoma (KC) and 90% of squamous cell carcinoma (SCC).^38^ In KC, pDCs were more abundant, representing a greater proportion of the inflammatory infiltrate, with greater activity, suggesting a possible role of pDCS in the pathogenesis of KC regression. Recently, Fraga et al. corroborated the abundant presence of pDCs in both KC and SCC, without significant differences.^39^

Imiquimod, a selective TLR7 agonist *via* transcription factor NF-kB activation and production of inflammatory cytokines, such as type 1 IFN, is used as treatment for some skin tumors.^40^ Basal cell carcinomas (BCCs) treated with imiquimod showed activated pDCs producing IFN-α in the peritumoral infiltrate.^41^ Subsequently, Ogawa et al. demonstrated correlation between the amount of pDCs recruited in actinic keratosis (AK) lesions and the therapeutic effect of imiquimod in these cases.^40^

The presence of pDCs has also been described in melanoma, mainly located around the vessels and close to tumor cells, both in primary cases and in metastases. However, these pDCs are not activated, there is a lack of TLR7 and TLR9 signaling in the tumor environment, there is no production of type 1 IFN, and they have been associated with tumor growth. In contrast, regressive melanomas have activated pDCs, indicating possible antitumor activity. Treatment of skin tumors (AK, superficial BCC, Bowen's disease and even lentigo maligna melanoma) with imiquimod (TLR7 and TLR9 agonist) activates the pDCs to produce type 1 IFN.^42^

Similarly, Karouni et al. described that pDCs are present in 90% of cases of Kaposi's sarcoma (KS), with reduced MxA expression, indicating suppression by Kaposi's sarcoma-associated herpesvirus (KSHV), through viral proteins that would inhibit the signaling pathways of pDCs activation.

Main findings of pDCs in skin diseases are summarized in Table 1.^43^

**Table 1:** Skin diseases and plasmacytoid dendritic cells (pDCs).

Dermatological diseases	pDCs
Lupus erythematosus	SLE: reduced circulating pDCs^3^, ^11^, ^12^, ^13^
	DL: large amounts of IFN-α producing pDCs^3^, ^11^, ^12^, ^13^
	SAL, DL: superficial distribution^3^
	TL, MHR: distribution around deep vessels^3^
	pDCs localized preferably in the epidermis in patients with DM and in the dermis in cases of lupus^11^
Lichen planus	pDCs arranged similarly to the band-like lymphocytic infiltrate at the dermo-epidermal junction^16^, ^17^
	pDCs increased in oral lichen planus^18^
Lichen striatus	pDCs present in the inflammatoryinfiltrate
	Perieccrine distribution of pDCs^19^
Atopic dermatitis	Increased circulating pDCs compared to controls
	Lower recruitment of pDCs in AD lesions compared to other dermatoses, such as lupus erythematosus and psoriasis^20^, ^21^
Polymorphic light eruption	Wackernagel et al.:^22^ absent pDCs
	Rossi et al.:^23^ increased pDCs in PLE lesions, mainly dermal. The authors attribute the difference to the methodology witch included of a photoprovocation testing.
Psoriasis	pDCs present in the T lymphocyte infiltrate in the dermis of psoriatic plaques and in the perilesional skin^24^
	Reduction of pDCs in the peripheral blood of patients with psoriasis^24^
	Heliotherapy: reduction of pDCs amount and MxA expression^25^
	Strong chemerin expression and presence of pDCs in the perilesional skin dermis^26^
Pityriasis lichenoides	Strong MxA expression in PL lesions (local production of type 1 IFN)^27^, ^28^
	Presence of pDCs on the skin in all cases (PLEVA and PLC)^29^
Graft *vs.* host disease	Presence of pDCs in skin fragments of patients with acute GVHD, strong expression of MxA, signaling local production of type 1 IFN^30^
	Reduction of serum pDCs in patients with acute GVHD^31^
	Presence of CD123+ cells in the oral mucosa of patients with chronic GVHD was not superior^32^
Warts	Saadeh et al.:^33^ activated pDCs in inflamed viral warts, contrary to what was found by Tomasini et al.^3^ (possible differences in histologic criteria for inflamed warts).
	Reduction in pDCs in peripheral blood and in skin lesions, absence of type 1 IFN production (MxA without expression) in patients with WHIM syndrome^34^
Fungal diseases	pDCs found in 37% of the skin fragments of patients with CBM and in 50% of the specimens from patients with PCM, but absent in cases of lobomycosis^35^
Leprosy	Massone et al.:^36^ absence of CD123 expression, except for focal expression in two cases of erythema nodosum leprosum
	Hirai et al.:^37^ pDCs in the inflammatory infiltrate and around the vessels. Higher number of pDCs in tuberculoid cases compared to virchowian cases.
Skin tumors	Keratoacanthoma: presence of pDCs in 100% of cases^38^
	SCC: presence of pDCs in 90% of cases^38^
	AK: correlation between the amount of pDCs recruited in the lesions during treatment with imiquimod and the therapeutic effect of the drug^40^
	BCC: presence of activated pDCs producing IFN-α in the peritumoral infiltrate during treatment with imiquimod^41^
	Melanoma: pDCs arranged around the vessels and close to the tumor cells, but not activated. Presence associated with tumor growth. pDCs activated in cases of regressive melanoma^42^
	KS: pDCs present in 90% of cases, but reduced expression of MxA (possible suppression of pDCs by KSHV)^43^

pDCs, plasmacytoid dendritic cells; SLE, systemic lupus erythematosus; DL, discoid lupus; SAL, subacute lupus; TL, tumid lupus; MRH, multicentric reticulohistiocytosis; DM, dermatomyositis; AD, atopic dermatitis; PLE, polymorphic light eruption; PL, pityriasis lichenoides; PLEVA, pityriasis lichenoides et varioliformis acuta; PLC, pityriasis lichenoides chronica; GVHD, graft *vs*. host disease; WHIM, warts, hypogammaglobulinemia, infections, and myelokathexis; CBM, chromoblastomycosis; PCM, paracoccidioidomycosis; SCC, squamous cell carcinoma; AK, actinic keratosis; BCC, basal cell carcinoma; KS, Kaposi's sarcoma; KSHV, Kaposi's sarcoma-associated herpesvirus.

## Conclusions

It is currently known that pDCs are the link between innate and adaptive immunity. These cells are not skin residents, and therefore their presence in the skin suggest a role (either central or adjuvant) in inflammatory, autoimmune, neoplastic and infectious processes. More studies are needed regarding such cells,which may lead to novel therapeutic targets.

## Financial support

This study was financed by FUNADERSP (Dermatology Support Fund of the State of São Paulo-Sebastião Sampaio) – Project 52-2017.

## Authors’ contributions

Natasha Favoretto Dias de Oliveira: Approval of the final version of the manuscript; design and planning of the study; drafting and editing of the manuscript; collection, analysis, and interpretation of data; critical review of the literature.

Claudia Giuli Santi: Approval of the final version of the manuscript; design and planning of the study; collection, analysis, and interpretation of data; effective participation in research orientation; critical review of the manuscript.

Celina Wakisaka Maruta: Approval of the final version of the manuscript; design and planning of the study; collection, analysis, and interpretation of data; effective participation in research orientation; critical review of the manuscript.

Valeria Aoki: Approval of the final version of the manuscript; design and planning of the study; collection, analysis, and interpretation of data; effective participation in research orientation; critical review of the literature; critical review of the manuscript.

## Conflicts of interest

None declared.
